# Functional and developmental changes in the inner hair cell ribbon synapses caused by Myosin VI knockout and deafness-inducing point mutation

**DOI:** 10.1038/s41420-023-01473-3

**Published:** 2023-05-31

**Authors:** Ning Yin, Jingjing Zhao, Panpan Zhang, Baofu Yu, Renjie Chai, Geng-Lin Li

**Affiliations:** 1 ENT Institute and Department of Otorhinolaryngology, Eye & ENT Hospital, Fudan University, Shanghai, 200031 China; 2 NHC Key Laboratory of Hearing Medicine, Fudan University, Shanghai, 200031 China; 3 State Key Laboratory of Medical Neurobiology and MOE Frontiers Center for Brain Science, Fudan University, Shanghai, 200031 China; 4 State Key Laboratory of Bioelectronics, Department of Otolaryngology Head and Neck Surgery, Zhongda Hospital, School of Life Sciences and Technology, Advanced Institute for Life and Health, Jiangsu Province High-Tech Key Laboratory for Bio-Medical Research, Southeast University, Nanjing, 210096 China; 5 Co-Innovation Center of Neuroregeneration, Nantong University, Nantong, 226001 China; 6 Department of Otolaryngology Head and Neck Surgery, Sichuan Provincial People’s Hospital, University of Electronic Science and Technology of China, Chengdu, China; 7 Department of Plastic and Reconstructive Surgery, Shanghai Ninth People’s Hospital Affiliated to Shanghai Jiaotong University School of Medicine, Shanghai, 200011 China

**Keywords:** Hair cell, Neurological disorders

## Abstract

Hearing loss is one of the most common neurosensory disorders in humans, and above half of hearing loss is caused by gene mutations. Among more than 100 genes that cause non-syndromic hearing loss, myosin VI (MYO6) is typical in terms of the complexity of underlying mechanisms, which are not well understood. In this study, we used both knock-out (*Myo6*^−/−^) and point mutation (*Myo6*^*C442Y*^) mice as animal models, performed whole-cell patch-clamp recording and capacitance measurement in the inner hair cells (IHCs) in the cochlea, and sought to reveal potential functional and developmental changes in their ribbon synapses. In *Myo6*^−*/*−^ cochleae of both before (P8-10) and after hearing onset (P18-20), exocytosis from IHCs, measured in whole-cell capacitance change (ΔC_m_), was significantly reduced, Ca^2+^ current amplitude (I_Ca_) was unchanged, but Ca^2+^ voltage dependency was differently altered, causing significant increase in Ca^2+^ influx in mature IHCs but not in immature IHCs. In immature IHCs of *Myo6*^*C442Y/C442Y*^ cochleae, neither ΔC_m_ nor I_Ca_ was altered, but both were reduced in mature IHCs of the same animal model. Furthermore, while the reduction of exocytosis was caused by a combination of the slower rate of depleting readily releasable (RRP) pool of synaptic vesicles and slower sustained release rate (SRR) in *Myo6*^−*/*−^ immature IHCs, it was likely due to smaller RRP and slower SRR in mature IHCs of both animal models. These results expand our understanding of the mechanisms of deafness caused by MYO6 mutations, and provide a solid theoretical and scientific basis for the diagnosis and treatment of deafness.

## Introduction

Of all the sensory systems in the human body, hearing is the most vulnerable to environmental and genetic factors, resulting in a decline in function or even a complete absence. According to the latest data published on the hereditary hearing loss website, 123 genes are known to have mutations or deletions that can lead to non-syndromic hearing loss [1]. In addition, mutations or deletions of more than 400 genes can lead to syndromic hearing loss [2]. It is worth mentioning that most of these genes contain multiple types of deafness mutations, and the mechanisms of these mutations are not the same [3, 4]. Therefore, although hereditary deafness research has received attention at an early stage, elucidating the deafness mechanism of different deafness genes seems to be a long way to go. Due to the unparalleled structural complexity and functional sophistication of the auditory pathway, especially the cochlea, we can only systematically study different types of mutations in different deafness genes one by one, and there is no shortcut.

Among all the genes that can cause non-syndromic deafness, MYO6 is a very typical representative in terms of the complexity of the deafness mechanism. MYO6 encodes Myosin VI, and the same family contains a large number of genes responsible for different intracellular transport and motor functions, including skeletal muscle contraction [5, 6]. Since the occurrence of hearing in the cochlea relies heavily on the physical property of cells, especially the hair bundles at the top of hair cells are composed mainly of actin, so far at least six members of myosin gene family, including MYO6 are thought to be associated with hearing [3]. Pathogenic variants in the MYO6 gene cause either autosomal dominant inherited non-syndromic hearing loss (DFNA22) or autosomal recessive inherited non-syndromic hearing loss (DFNB37) [7, 8]. MYO6 was first associated with hearing thanks to a new recessive gene mutation found in Jackson’s laboratory in the 1960s, which can lead to a serious decline in the hearing and balance perception functions of mice. The gene mutation named Snell’s waltzer leads to the deletion of Myosin VI [9]. Roux et al. found that the amplitude of calcium current in IHCs of adult Snell’s waltzer mice was not significantly different from that of wild-type mice, but the synaptic vesicle release was remarkably reduced [10]. Strangely, on the same Snell’s waltzer mice model, another study presented the opposite conclusion: the amplitude of calcium current in IHCs decreased, but there was no significant difference in vesicle release compared with wild-type mice [11]. Therefore, it is necessary to systematically and carefully analyze the functional changes of synaptic transmission of IHCs on different MYO6 mutation mice models in order to fully and deeply reveal the deafness mechanism of Myosin VI gene mutation.

The MYO6 p.C442Y mutation causes DFNA22. Carriers of the MYO6 p.C442Y mutation begin to develop progressive hearing loss during childhood and show profound sensorineural hearing loss by middle age [7]. *Myo6*^*C442Y*^ mutation mice with a semidominant inheritance pattern exhibits hearing loss starting from 3 weeks after birth and progresses to severe deafness accompanied by degeneration of hair cells and disorganization of the stereocilia in the organ of Corti [12].

In this study, we used *Myo6*^*C442Y*^ point mutation mice and MYO6 knock-out mice as experimental animals, combined with molecular biology and whole-cell patch clamp technology to explore the physiological and pathological mechanisms of MYO6 gene mutation or deletion on auditory development and mutation-induced deafness, thus providing a certain experimental basis for improving and treating non-syndromic deafness.

## Results

### Effects of MYO6 knock-out on synaptic transmission in developing mice IHCs

We studied synaptic transmission in IHCs of MYO6 knock-out developing mice using whole-cell patch-clamp recordings. IHCs depolarization leads to Ca^2+^ entry, triggering an increase in the overall Ca^2+^ inflow and instantaneous Cm increments, which is controlled by activation of the voltage-dependent Ca^2+^ current.

**Fig. 1:**
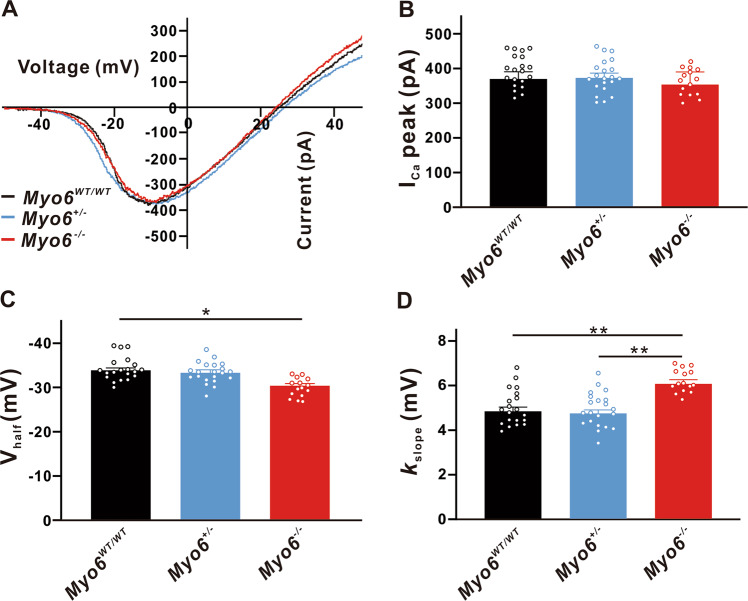
Properties of I_Ca_ in immature IHCs. **A** Representative I-V curve of Ca^2+^ currents recorded from *Myo6*^*WT/WT*^ mice (black), *Myo6*^*+/*−^ mice (blue) and *Myo6*^−*/*−^ mice (red) IHCs at P8-10, induced by a voltage ramp from −90 to +70 mV under voltage-clamp and then leak subtracted. **B** The peak amplitude of Ca^2+^ current (I_Ca_) from three genotypes immature mice IHCs has no significant difference. **C**, **D** I_Ca_ in *Myo6*^−*/*−^ mice IHCs has a less negative half-activation voltage (V_half_) than *Myo6*^*WT/WT*^ and a bigger activation slope (*k*_slope_) than *Myo6*^*WT/WT*^ and *Myo6*^*+/*−^. Data are presented as the mean ± SEM. Statistical analysis was by one-way ANOVA. * means *P* < 0.05 and ** means *P* < 0.01.

Three groups of wild (*Myo6*^*WT/WT*^), heterozygous (*Myo6*^*+/*−^) and homozygous (*Myo6*^−*/*−^) mice for experiments to analyze the genetic characteristics of different functional indicators. The acutely isolated mice basement membranes were infiltrated in the external fluid of calcium currents. IHCs were hold at −90 mV and then applied a slow-rising ramp voltage stimulation from −90 mV to +70 mV to induce whole-cell calcium currents at different voltages, and further analyzing the calcium currents at different voltages can obtain the I-V curve (Fig. 1A). We found that there was no significant difference in the peak amplitude of I_Ca_ in IHCs from three genotypes developing mice (*Myo6*^*WT/WT*^: 369.5 ± 20.89 pA, *n* = 20; *Myo6*^*+/*−^: 372.9 ± 13.86 pA, *n* = 21, *P* > 0.05 vs. *Myo6*^*WT/WT*^; *Myo6*^−*/*−^: 353.5 ± 36.48 pA, *n* = 15, *P* > 0.05 vs. *Myo6*^*WT/WT*^) (Fig. 1B). Then we use Boltzmann function fitting the current-voltage curve to obtain V_half_ and *k*, which depict the steepness of voltage dependence in Ca^2+^ channels activation in order to characterize the functional properties of IHCs from the three different genotypes mice more comprehensively. V_half_ describes the membrane potential where the conductance is half activated, while *k* reveals the voltage sensitivity of the activation. Noticing I_Ca_ in *Myo6*^−*/*−^ mice has a less negative V_half_ than *Myo6*^*WT/WT*^ (*Myo6*^*WT/WT*^: −33.89 ± 0.524 mV, *n* = 20; *Myo6*^−*/*−^: −30.39 ± 0.5002 mV, *n* = 15, *P* < 0.05) and a bigger activation slope (*k*_slope_) than *Myo6*^*WT/WT*^ and *Myo6*^*+/*−^ (*Myo6*^*WT/WT*^: 4.817 ± 0.1977 mV, *n* = 20; *Myo6*^*+/*−^: 4.726 ± 0.1597 mV, *n* = 21; *Myo6*^−*/*−^: 6.050 ± 0.185 mV, *n* = 15, *P* < 0.01 vs. *Myo6*^*WT/WT*^ and *Myo6*^*+/*−^) (Fig. 1C, D).

**Fig. 2:**
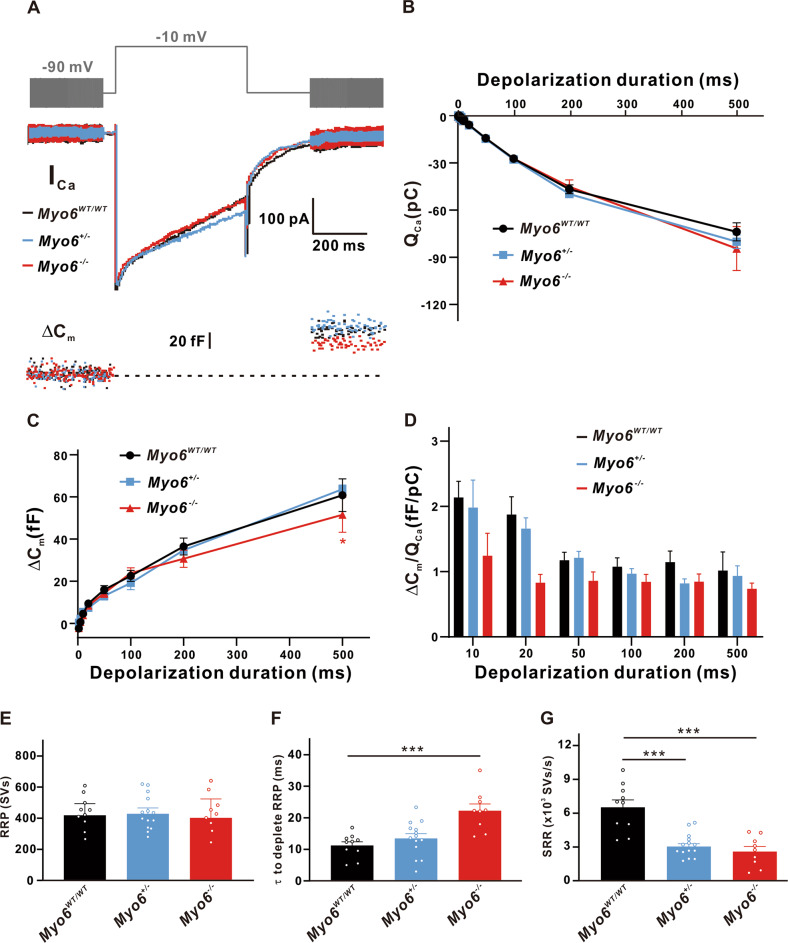
Exocytosis from immature IHCs. **A** A representative diagram of I_Ca_ and the corresponding ΔC_m_ in *Myo6*^*WT/WT*^ mice (black), *Myo6*^*+/*−^ mice (blue) and *Myo6*^−*/*−^ mice (red) IHCs at P8-10 of stimulation duration 500 ms. **B**–**D** Ca^2+^ influx (Q_Ca_) (**B**) and Ca^2+^ efficiency in triggering exocytosis, the ratio of ΔC_m_/Q_Ca_ (**D**) recorded from three genotypes immature mice IHCs in response to increasing stimulation durations from 2 ms to 500 ms both have no apparent change, but the ΔC_m (C) in_ *Myo6*^−*/*−^ mice IHCs is remarkably smaller than that in *Myo6*^*WT/WT*^ mice for duration 500 ms. **E** There was no obvious difference in the readily releasable pool (RRP) of synaptic vesicles. **F** The time constants (τ) to deplete RRP from *Myo6*^−*/*−^ mice IHCs was significantly longer than that from *Myo6*^*WT/WT*^ mice IHCs. **G** Sustained release rate (SRR) of synaptic vesicles from *Myo6*^−*/*−^ mice and *Myo6*^*+/*−^ mice IHCs was significantly slower than that from *Myo6*^*WT/WT*^ mice IHCs. Data are presented as the mean ± SEM. Statistical analysis was by one-way or two-way ANOVA. * means *P* < 0.05 and *** means *P* < 0.001.

Exocytosis results in incorporation of the membrane from synaptic vesicles into the cell membrane and therefore increases the cell membrane, which can be measured through whole-cell capacitance measurement [13]. Therefore, while recording the voltage-gated calcium current, whole-cell membrane capacitance change (ΔC_m_) was monitored to quantify the synaptic vesicle release of IHCs. Figure 2A shows a representative diagram of I_Ca_ and the corresponding ΔC_m_ in *Myo6*^*WT/WT*^, *Myo6*^*+/*−^ and *Myo6*^−*/*−^ developing mice IHCs of duration 500 ms. We found that there was no significant difference of Ca^2+^ influx (Q_Ca_) in IHCs from the three genotypes developing mice when pooled data for Ca^2+^ charge over all stimulation durations were evaluated (*n* = 10 in three groups, *P* > 0.05, Fig. 2B). Additionally, IHCs from *Myo6*^−*/*−^ (51.54 ± 8.258 fF, *n* = 10) released fewer synaptic vesicles for long stimulation compared with the *Myo6*^*WT/WT*^ (60.82 ± 7.798 fF, *n* = 10, *P* < 0.05; Fig. 2C). However, no observable difference was found in the Ca^2+^ efficiency of exocytosis (qualitatively defined as ΔC_m_ /Q_Ca_) for any three groups comparison of the mean values in response to all stimulations from 2 to 500 ms (Fig. 2D), suggesting that Ca^2+^ influx had similar efficiency in triggering *Myo6*^*WT/WT*^, *Myo6*^*+/*−^ and *Myo6*^−*/*−^ mice IHCs exocytosis.

Then we traced vesicles release through capacitance changes and identified two dynamic components of the vesicle pool. The initial small component of ΔC_m_ increases in response to a short stimulation, representing exocytosis of the RRP of synaptic vesicles located in the active region. Immediately afterward, ΔC_m_ continues to improve at a slower rate during the stimulation duration of up to 500 ms, indicating vesicles are released from the refilling-pool located farther away from the Ca^2+^ channel. Further analysis showed that the MYO6 knock-out had no effects on the RRP (*Myo6*^*WT/WT*^: 418.7 ± 74.99 SVs, *n* = 10; *Myo6*^*+/*−^: 428.3 ± 37.83 SVs, *n* = 14; *Myo6*^−*/*−^: 402.7 ± 120.9 mV, *n* = 9; *P* > 0.05), but obviously increased the time constants (τ) to deplete RRP (*Myo6*^*WT/WT*^: 11.23 ± 1.185 ms, *n* = 10; *Myo6*^*+/*−^: 13.50 ± 1.471 ms, *n* = 14; *Myo6*^−*/*−^: 22.24 ± 2.144 ms, *n* = 9, *P* < 0.001 vs. *Myo6*^*WT/WT*^) and effectively suppressed the sustained release rate (SRR) (*Myo6*^*WT/WT*^: 6 517 ± 1 263 SVs/s, *n* = 10; *Myo6*^*+/*−^: 3 041 ± 250.1 SVs/s, *n* = 14, *P* < 0.001 vs. *Myo6*^*WT/WT*^; *Myo6*^−*/*−^: 2 596 ± 642.5 SVs/s, *n* = 9, *P* < 0.001 vs. *Myo6*^*WT/WT*^) (Fig. 2E–G), suggesting that MYO6 knock-out developing mice replenish synaptic vesicles less efficiently.

### Effects of MYO6 knock-out on synaptic transmission in mature mice IHCs

**Fig. 3:**
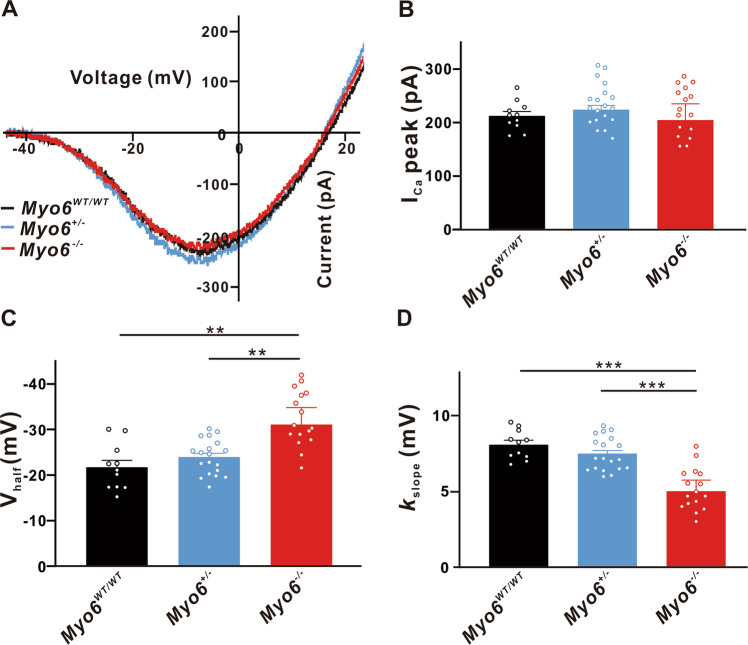
Properties of I_Ca_ in mature IHCs. **A** Representative I-V curve of Ca^2+^ currents recorded from *Myo6*^*WT/WT*^ mice (black), *Myo6*^*+/*−^ mice (blue) and *Myo6*^−*/*−^ mice (red) IHCs at P18-20. **B** The peak amplitude of Ca^2+^ current (I_Ca_) from three genotypes mature mice IHCs has no significant difference. **C**, **D** I_Ca_ in *Myo6*^−*/*−^ mice has a more negative half-activation voltage (V_half_) and a steeper activation slope (*k*_slope_) than *Myo6*^*WT/WT*^ and *Myo6*^*+/*−^. Data are presented as the mean ± SEM. Statistical analysis was by one-way ANOVA. ** means *P* < 0.01 and *** means *P* < 0.001.

We selected three groups of mature mice, *Myo6*^*WT/WT*^, *Myo6*^*+/*−^ and *Myo6*^−*/*−^ to record the Ca^2+^ currents of IHCs. Figure 3A shows that the I-V curve of Ca^2+^ currents was obtained. We found that the peak amplitude of I_Ca_ in IHCs from *Myo6*^*+/*−^ (224.0 ± 8.070 pA, *n* = 19, *P* > 0.05) and *Myo6*^−*/*−^ mice (204.6 ± 30.43 pA, *n* = 16, *P* > 0.05) has no significant change compared with that of *Myo6*^*WT/WT*^ mice (212.5 ± 8.116 pA, *n* = 11) (Fig. 3B). Figure 3C, D show that I_Ca_ in *Myo6*^−*/*−^ mice IHCs has a more negative V_half_ (−31.11 ± 3.688 mV, *n* = 16, *P* < 0.01 vs. *Myo6*^*WT/WT*^ and *Myo6*^*+/*−^) and a steeper activation slope (5.016 ± 0.7266 mV, *n* = 16, *P* < 0.001 vs. *Myo6*^*WT/WT*^ and *Myo6*^*+/*−^) than *Myo6*^*WT/WT*^ (V_half_: −21.71 ± 1.513 mV, *n* = 11; *k*_slope_: 8.091 ± 0.2885 mV, *n* = 11) and *Myo6*^*+/*−^ (V_half_: −24.00 ± 0.7672 mV, *n* = 19; *k*_slope_: 7.504 ± 0.1932 mV, *n* = 19), revealing that under physiological conditions calcium channels may cause more Ca^2+^ to flow into the IHCs of *Myo6*^−*/*−^ mice.

**Fig. 4:**
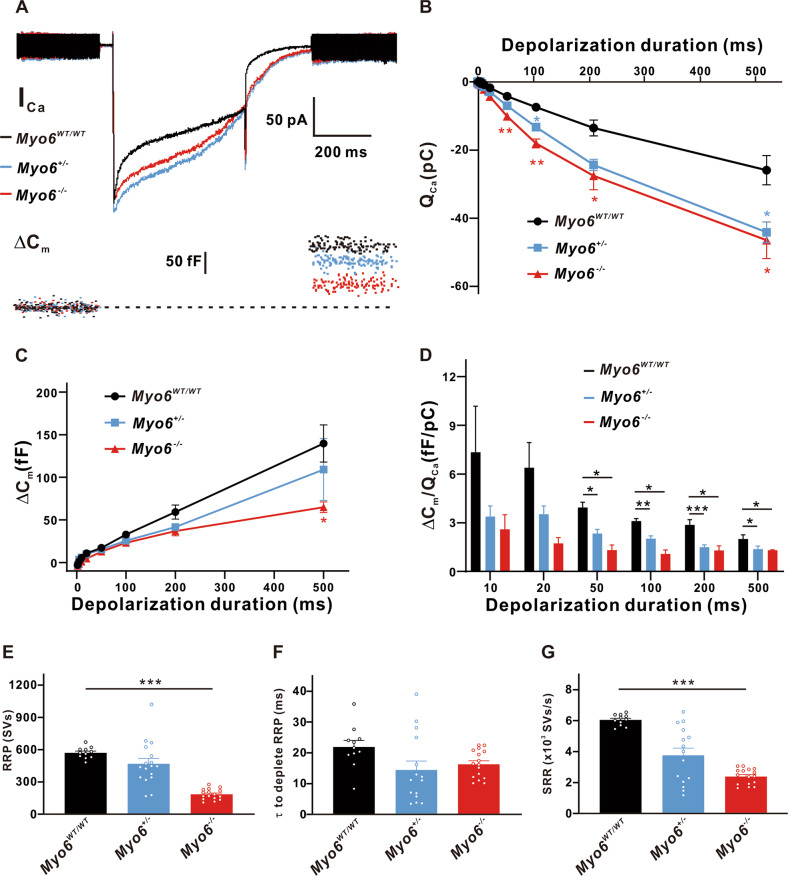
Exocytosis from mature IHCs. **A** A representative diagram of I_Ca_ and the corresponding ΔC_m_ in *Myo6*^*WT/WT*^ mice (black), *Myo6*^*+/*−^ mice (blue) and *Myo6*^−*/*−^ mice (red) IHCs at P18-20 of stimulation duration 500 ms. **B**–**D** Q_Ca_ (**B**) and the ratio of ΔC_m_/Q_Ca_ (**D**) recorded from *Myo6*^*+/*−^ and *Myo6*^−*/*−^ mice IHCs have significant difference than that of *Myo6*^*WT/WT*^ mice for both short and long stimulation, and the ΔC_m_ (C) in *Myo6*^−*/*−^ mice IHCs is remarkably smaller than that in *Myo6*^*WT/WT*^ mice for duration 500 ms. **E** RRP from *Myo6*^−*/*−^ mice IHCs was prominently smaller than that from *Myo6*^*WT/WT*^ mice IHCs. **F** There is no obvious difference inτto deplete RRP from three genotypes mature mice. **G** SRR of synaptic vesicles from *Myo6*^−*/*−^ mice IHCs was significantly slower than that from *Myo6*^*WT/WT*^ mice IHCs. Data are presented as the mean ± SEM. Statistical analysis was by one-way or two-way ANOVA. * means *P* < 0.05, ** means *P* < 0.01 and *** means *P* < 0.001.

A representative diagram of I_Ca_ and the corresponding ΔC_m_ in *Myo6*^*WT/WT*^, *Myo6*^*+/*−^ and *Myo6*^−*/*−^ mature mice IHCs of duration 500 ms is shown in Fig. 4A. Further research showed more Ca^2+^ influx (Q_Ca_) in IHCs from *Myo6*^*+/*−^ and *Myo6*^−*/*−^ mice (*n* = 12 in three groups, *P* < 0.05 ∼ 0.01 vs. *Myo6*^*WT/WT*^) (Fig. 4B). Moreover, IHCs from *Myo6*^−*/*−^ mice (64.94 ± 6.344 fF, *n* = 12) released fewer synaptic vesicles for 500 ms stimulation compared with the *Myo6*^*WT/WT*^ (139.7 ± 21.88 fF, *n* = 12, *P* < 0.05; Fig. 4C). Figure 4D manifests the value of ΔC_m_/Q_Ca_ in *Myo6*^*+/*−^ and *Myo6*^−*/*−^ mice IHCs is less than that of *Myo6*^*WT/WT*^ mice for both short and long stimulation (n = 12 in three groups, *P* < 0.05 ∼ 0.001), indicating that the Ca^2+^ efficiency of triggering synaptic vesicles release in mature MYO6 knock-out mice was lower than that in wild-type mice. Then we observed that the RRP and SRR in IHCs from *Myo6*^−*/*−^ mice both prominently smaller than that in IHCs from *Myo6*^*WT/WT*^ mice (RRP: 570.7 ± 15.78 SVs of *Myo6*^*WT/WT*^ mice, *n* = 11; 184.4 ± 12.30 SVs of *Myo6*^−*/*−^ mice, *n* = 16, *P* < 0.001; SRR: 6 046 ± 291.4 SVs/s of *Myo6*^*WT/WT*^ mice, *n* = 11; 2 387 ± 255.9 SVs/s of *Myo6*^−*/*−^ mice, *n* = 16, *P* < 0.001), suggesting that MYO6 knock-out mature mice have a smaller readily releasable pool of synaptic vesicles and replenish synaptic vesicles less efficiently which manifesting its poor function of exocytosis (Fig. 4E, G).

### Effects of *Myo6*^*C442Y*^ point mutation on synaptic transmission in developing mice IHCs

Three groups of wild (*Myo6*^*WT/WT*^), heterozygous (*Myo6*^*C442Y/WT*^) and homozygous (*Myo6*^*C442Y/C442Y*^) mice were selected for experiments to analyze the genetic characteristics of different functional indicators. We first investigated developing mice.

**Fig. 5:**
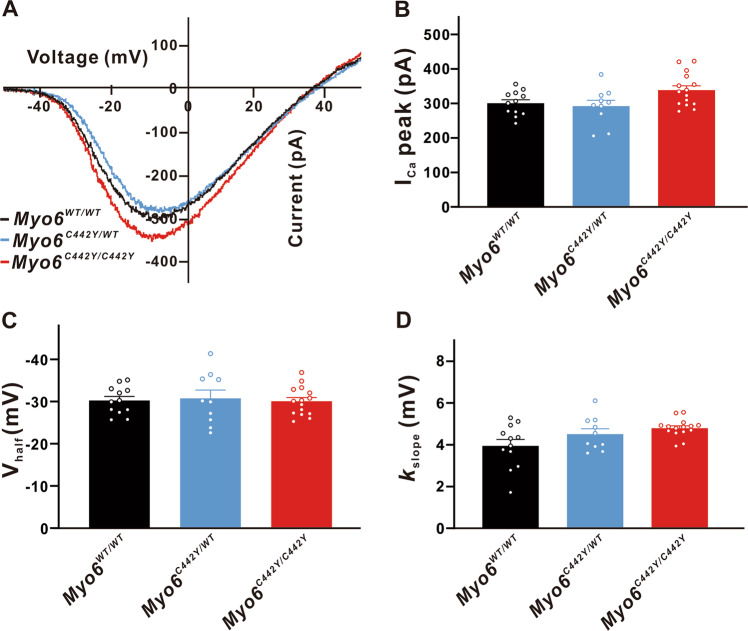
Properties of I_Ca_ in developing IHCs. **A** Representative I-V curve of Ca^2+^ currents recorded from *Myo6*^*WT/WT*^ mice (black), *Myo6*^*C442Y/WT*^ mice (blue) and *Myo6*^*C442Y/C442Y*^ mice (red) IHCs at P8-10. **B**–**D** I_Ca_ (**B**), V_half_ (**C**) and *k*_slope_ (**D**) from three genotypes developing mice IHCs have no distinct difference. Data are presented as the mean ± SEM. Statistical analysis was by one-way ANOVA. *P* > 0.05.

*Myo6*^*C442Y*^ point mutation had no obvious effects on the Ca^2+^ currents amplitude (*Myo6*^*WT/WT*^: 300.5 ± 25.32 pA, *n* = 12; *Myo6*^*C442Y/WT*^: 292.2 ± 26.59 pA, *n* = 10, *P* > 0.05 vs. *Myo6*^*WT/WT*^; *Myo6*^*C442Y/C442Y*^: 338.7 ± 24.37 pA, *n* = 15, *P* > 0.05 vs. *Myo6*^*WT/WT*^), V_half_ (*Myo6*^*WT/WT*^: -30.26 ± 2.337 mV, *n* = 12; *Myo6*^*C442Y/WT*^: -30.79 ± 2.455 mV, *n* = 10, *P* > 0.05 vs. *Myo6*^*WT/WT*^; *Myo6*^*C442Y/C442Y*^: -30.09 ± 2.250 mV, *n* = 15, *P* > 0.05 vs. *Myo6*^*WT/WT*^) and slope factor (*k*_slope_) of IHCs (*Myo6*^*WT/WT*^: 3.951 ± 0.4046 mV, *n* = 12；*Myo6*^*C442Y/WT*^: 4.512 ± 0.4249 mV, *n* = 10, *P* > 0.05 vs. *Myo6*^*WT/WT*^; *Myo6*^*C442Y/C442Y*^: 4.797 ± 0.3895 mV, *n* = 15, *P* > 0.05 vs. *Myo6*^*WT/WT*^) (Fig. 5A–D).

**Fig. 6:**
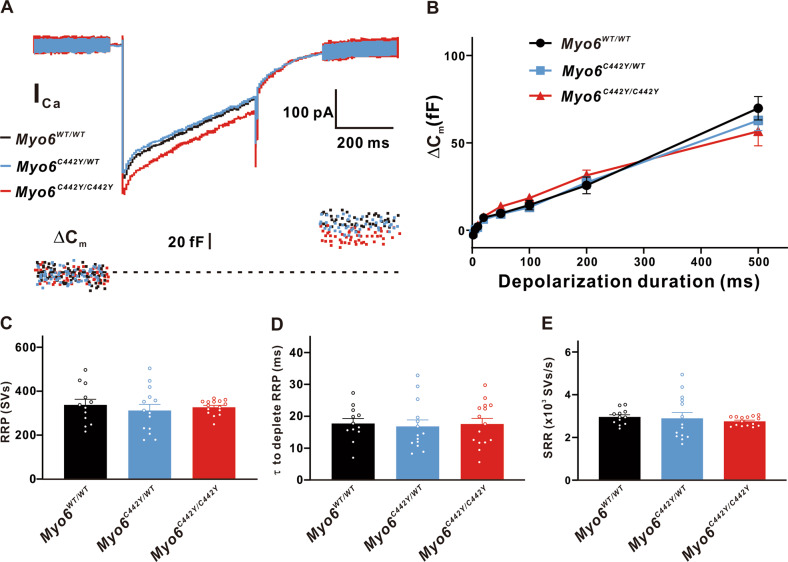
Exocytosis from developing IHCs. **A** A representative diagram of I_Ca_ and the corresponding ΔC_m_ in *Myo6*^*WT/WT*^ mice (black), *Myo6*^*C442Y/WT*^ mice (blue) and *Myo6*^*C442Y/C442Y*^ mice (red) IHCs at P8-10 of stimulation duration 500 ms. **B**–**E** ΔC_m (B),_ RRP (**C**), τ to deplete RRP (**D**) and SRR (**E**) in *Myo6*^*C442Y*^ point mutation mice IHCs have no remarkable change than that in *Myo6*^*WT/WT*^ mice under physiological conditions. Data are presented as the mean ± SEM. Statistical analysis was by one-way or two-way ANOVA. *P* > 0.05.

And under our experimental conditions we found that the Ca^2+^-induced exocytosis of IHCs in the developing mice was also not affected by the *Myo6*^*C442Y*^ point mutation, ΔC_m_ (*n* = 10 in three groups, *P* > 0.05 vs. Myo6^WT/WT^), RRP (Myo6^WT/WT^: 337.8 ± 56.66 SVs, *n* = 12; Myo6^C442Y/WT^: 312.5 ± 32.50 SVs, *n* = 14, *P* > 0.05 vs. *Myo6*^*WT/WT*^; *Myo6*^*C442Y/C442Y*^: 327.0 ± 25.88 SVs, *n* = 15, *P* > 0.05 vs. *Myo6*^*WT/WT*^), SRR (*Myo6*^*WT/WT*^: 2 961 ± 176.3 SVs/s, n = 12; *Myo6*^*C442Y/WT*^: 2 894 ± 410.7 SVs/s, *n* = 14, *P* > 0.05 vs. *Myo6*^*WT/WT*^; *Myo6*^*C442Y/C442Y*^: 2 755 ± 148.1 SVs/s, *n* = 15, *P* > 0.05 vs. *Myo6*^*WT/WT*^), and τ to deplete RRP (*Myo6*^*WT/WT*^: 17.73 ± 3.389 ms, *n* = 12; *Myo6*^*C442Y/WT*^:16.82 ± 3.866 ms, *n* = 14, *P* > 0.05 vs. *Myo6*^*WT/WT*^; *Myo6*^*C442Y/C442Y*^: 17.56 ± 5.322 ms, *n* = 15, *P* > 0.05 *vs*. *Myo6*^*WT/WT*^) (Fig. 6A–E).

### Effects of *Myo6*^*C442Y*^ point mutation on synaptic transmission in mature mice IHCs

**Fig. 7:**
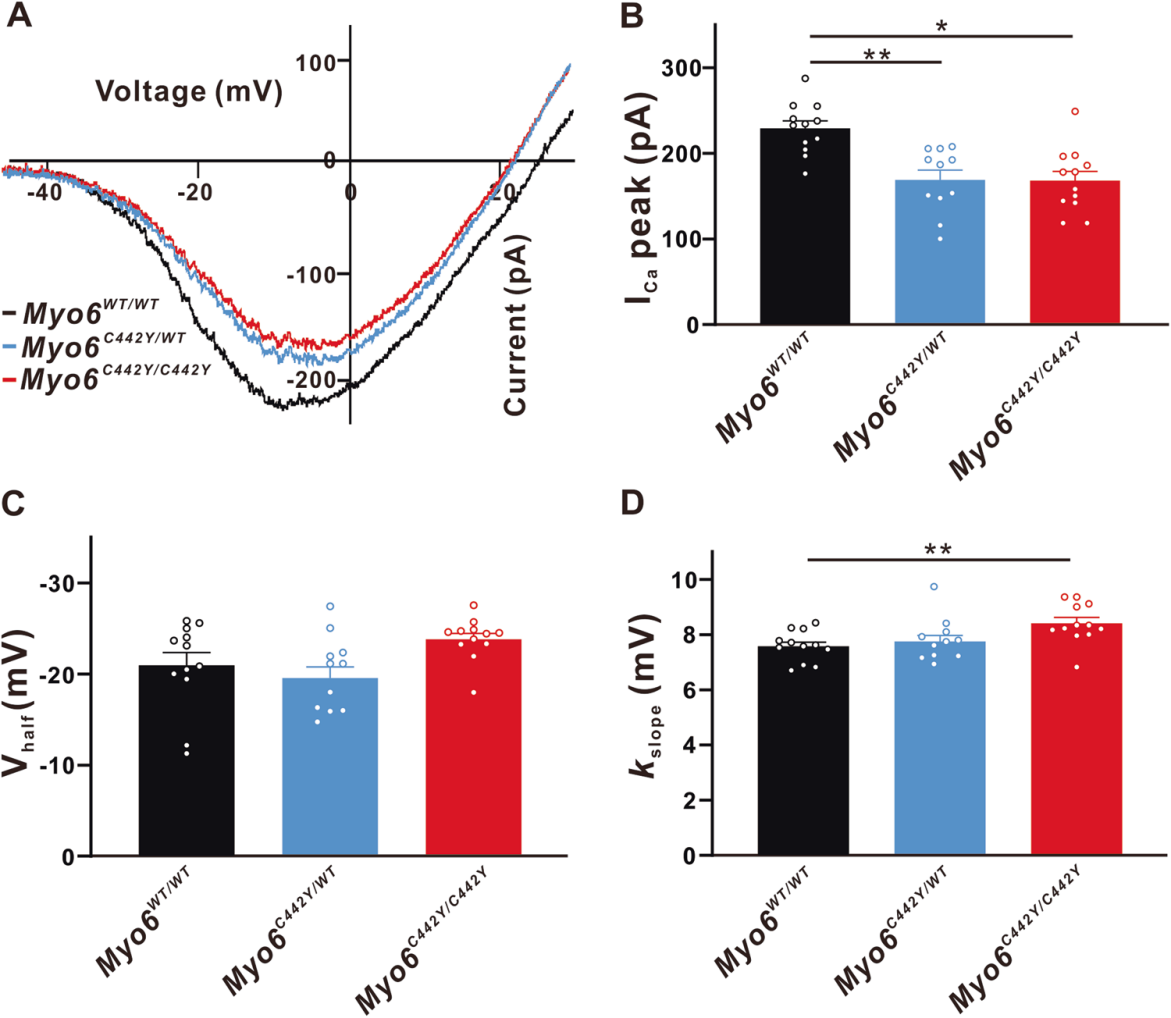
Properties of I_Ca_ in mature IHCs. **A** Representative I-V curve of Ca^2+^ currents recorded from *Myo6*^*WT/WT*^ mice (black), *Myo6*^*C442Y/WT*^ mice (blue) and *Myo6*^*C442Y/C442Y*^ mice (red) IHCs at P18-20. **B** *Myo6*^*C442Y*^ point mutation mice IHCs have smaller I_Ca_ amplitude than wild-type mice. **C**, **D** I_Ca_ in *Myo6*^*C442Y/C442Y*^ mice IHCs has a similar V_half_ and a bigger *k*_slope_ compared to *Myo6*^*WT/WT*^ mice. Data are presented as the mean ± SEM. Statistical analysis was by one-way ANOVA. * means *P* < 0.05 and ** means *P* < 0.01.

Moreover, *Myo6*^*WT/WT*^, *Myo6*^*C442Y/WT*^ and *Myo6*^*C442Y/C442Y*^ mature mice were selected to improve our research. We found that the *Myo6*^*C442Y*^ mutation resulted in a decrease in I_Ca_ amplitude of IHCs (*Myo6*^*WT/WT*^: 229.2 ± 18.65 pA, *n* = 12; *Myo6*^*C442Y/WT*^:169.0 ± 20.45 pA, *n* = 11, *P* < 0.01 vs. *Myo6*^*WT/WT*^; *Myo6*^*C442Y/C442Y*^: 168.1 ± 20.40 pA, *n* = 12; *P* < 0.05 vs. *Myo6*^*WT/WT*^) (Fig. 7A, B). But there was no obviously difference in V_half_ of I_Ca_ (*Myo6*^*WT/WT*^: -20.96 ± 1.702 mV, *n* = 12; *Myo6*^*C442Y/WT*^: −19.56 ± 1.903 mV, *n* = 11, *P* > 0.05 vs. *Myo6*^*WT/WT*^; *Myo6*^*C442Y/C442Y*^: -23.83 ± 1.903 mV, *n* = 12, *P* > 0.05 vs. *Myo6*^*WT/WT*^) (Fig. 7C). Figure 7D testifies to the slope factor of *Myo6*^*C442Y/C442Y*^ mice IHCs (8.410 ± 0.201 mV, *n* = 12) is bigger than that of *Myo6*^*WT/WT*^ (7.572 ± 0.213 mV, *n* = 12; *P* < 0.01), indicative of less Ca^2+^ influx the IHCs of *Myo6*^*C442Y*^ point mutation mice induced by Ca^2+^ channels.

**Fig. 8:**
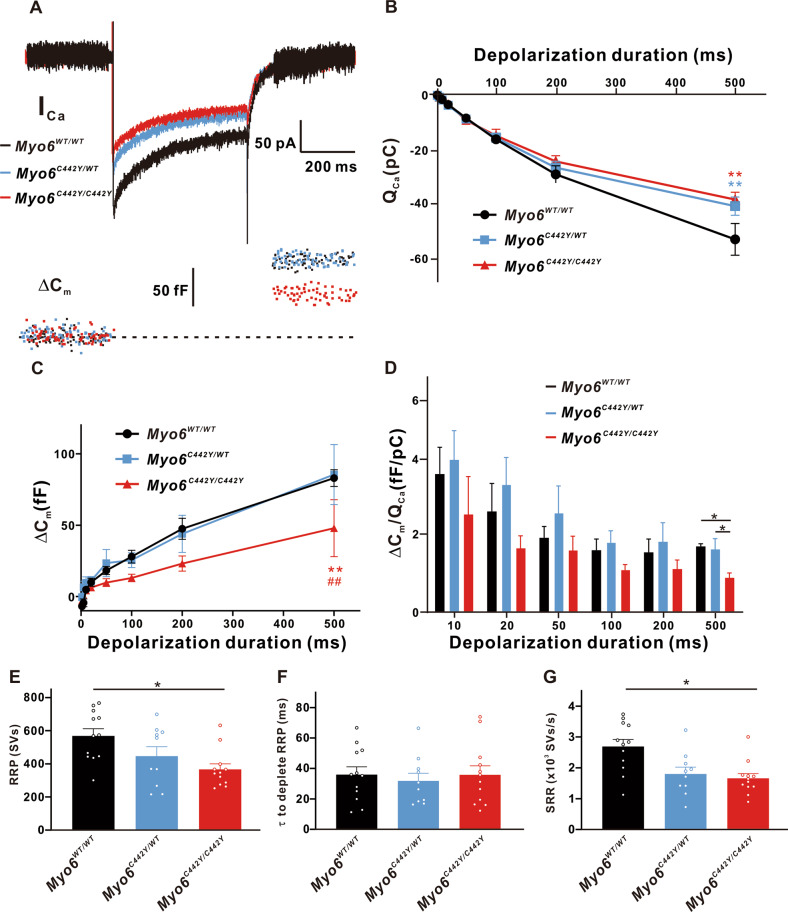
Exocytosis from mature IHCs. **A** A representative diagram of I_Ca_ and the corresponding ΔC_m_ in *Myo6*^*WT/WT*^ mice (black), *Myo6*^*C442Y/WT*^ mice (blue) and *Myo6*^*C442Y/C442Y*^ mice (red) IHCs at P18-20 of stimulation duration 500 ms. **B** Q_Ca_ in *Myo6*^*C44Y2/WT*^ and *Myo6*^*C442Y/C442Y*^ mice IHCs are significant difference than that of *Myo6*^*WT/WT*^ mice for duration 500 ms. **C**, **D** ΔC_m_ (**C**) and the ratio of ΔC_m_/Q_Ca_ (**D**) recorded from *Myo6*^*C442Y/C442Y*^ mice IHCs are remarkable down-regulation than that from *Myo6*^*WT/WT*^ and *Myo6*^*C44Y2/WT*^ mice of duration 500 ms. **E**–**G** RRP (E) and SRR (**G**) from *Myo6*^*C442Y/C442Y*^ mice IHCs are obviously smaller than that from *Myo6*^*WT/WT*^ mice IHCs, but there is no distinct change inτto deplete RRP from three genotypes mature mice IHCs. Data are presented as the mean ± SEM. Statistical analysis was by one-way or two-way ANOVA. * means *P* < 0.05, ** means *P* < 0.01, ^##^ means *P* < 0.01.

Then we varied stimulation duration from 2 to 500 ms, founding less Q_Ca_ in IHCs from *Myo6*^*C442Y/WT*^ and *Myo6*^*C442Y/C442Y*^ mice (*n* = 12 in three groups, *P* < 0.01 vs. *Myo6*^*WT/WT*^) (Fig. 8B). Giving 500 ms stimulation, the induced ΔC_m_ of *Myo6*^*C442Y/C442Y*^ mice (47.90 ± 10.96 fF, *n* = 12) was remarkably less than that of *Myo6*^*WT/WT*^ mice (82.95 ± 10.45 fF, *n* = 12, *P* < 0.01) and *Myo6*^*C442Y/WT*^ mice (85.4 ± 10.96 fF, *n* = 12, *P* < 0.01) (Fig. 8C). And the Ca^2+^ efficiency of triggering synaptic vesicles release in mature *Myo6*^*C442Y/C442Y*^ mice (0.899 ± 0.151, *n* = 12) was lower than that in *Myo6*^*C442Y/WT*^ mice (1.172 ± 0.26, *n* = 12, *P* < 0.05) and *Myo6*^*WT/WT*^ mice (1.636 ± 0.299, *n* = 12, *P* < 0.05) (Fig. 8D). Figure 8E, G show that the RRP and SRR in IHCs from *Myo6*^*C442Y/C442Y*^ mice both obviously decrease than that in IHCs from *Myo6*^*WT/WT*^ mice (RRP: 568.4 ± 56.23 SVs of *Myo6*^*WT/WT*^ mice, *n* = 12; 366.4 ± 36.29 SVs of *Myo6*^*C442Y/C442Y*^ mice, *n* = 12, *P* < 0.05; SRR: 2 691 ± 332.1 SVs/s of *Myo6*^*WT/WT*^ mice, *n* = 12; 1 658 ± 188.2 SVs/s of *Myo6*^*C442Y/C442Y*^ mice, *n* = 12, *P* < 0.05), testifying that *Myo6*^*C442Y*^ point mutation mature mice IHCs have comparative weak function of exocytosis.

## Discussion

MYO6 was one of the earliest deafness genes identified [14]. In cochlear hair cells it has been suggested that MYO6 could be involved in anchoring their apical membrane to the underlying actin-rich cuticular plate and also in the intracellular transport of synaptic vesicles and basolateral membrane proteins required for the functional maturation of IHCs at the onset of hearing [11, 15]. Mutations in the gene encoding myosin VI have been associated with dominant progressive and recessive congenital deafness in humans [7, 8]. The extraordinary sensitivity and timing accuracy of the auditory system depend on the exocytosis of the hair cell ribbon synapses, which work at the most appropriate frequency. Therefore, we comprehensively reveal the deafening mechanism of MYO6 mutation by analyzing the synaptic transmission function of inner hair cells on different mouse models systematically. In the present study, we studied ribbon synapse functions in immature and mature IHCs of both *Myo6*^−*/*−^ and *Myo6*^*C442Y/C442Y*^ mice, allowing us to compare and contrast MYO6 functions over different developmental stages and different mutants. Our study revealed the complexity of MYO6 functions in hearing, which is valuable towards building a whole and complete understanding of hearing deficits in DFNA22 and DFNB37.

It is reported that MYO6 exists in the active region of IHC synapses, and its absence prevents the normal maturation of IHC ribbon synapses [10]. We found that MYO6 deletion did not change the I_Ca_ amplitude of IHCs, but lead to a markedly reduced synaptic exocytosis of IHCs both in immature and mature mice (Figs. 1B, 2C, 3B and 4C). The role of MYO6 in IHCs exocytosis is consistent with several studies. Roux et al. found that compared with wild-type mice, the amplitude of Ca^2+^ currents on the IHCs of adult Snell’s waltzer mutant mice has no obvious difference, but the vesicle release was significantly reduced [10]. MYO6 is necessary for efficient secretion and maintenance of Golgi apparatus structure [16]. This is also manifested in synaptic vesicle exocytosis of neuronal synapses [17]. In hippocampal neurons, both spontaneous and induced synaptic vesicle exocytosis were reduced by 35% in Snell’s waltzer mice [18]. And studies have shown that the defect of Ca^2+^- induced exocytosis is partial and only appears in mature IHCs absence of MYO6 [10], which consistent with our results (Figs. 2D and 4D). The reduced synaptic exocytosis observed in mature MYO6 knock-out mice IHCs may in principle be due to failure of IHC synaptic development, defective vesicle transport, inefficient Ca^2+^- exocytosis coupling, or a combination of these defects. On the contrary, on the same Snell’s waltzer mice model, the amplitude of Ca^2+^ currents in IHCs decreased, but there was no significant difference in vesicle release compared with wild-type mice [11].

It is worth noticing that at least two kinetically distinct components of vesicle release were in mammalian hair cells: a rapidly small RRP and a slower but larger SRP, and RRP depletion is discussed as a mechanism for fast auditory adaptation. Our results indicated that both MYO6 knock-out developing mice and mature mice replenish synaptic vesicles less efficiently, which manifesting the poor function of exocytosis (Figs. 2E–G and 4E–G). The reason for these in the IHCs, the main source for IHCs synaptic vesicle pool replenishment is endocytosis at the synaptic active zone and the peri-cuticular necklace [19, 20]. And the abundance of MYO6 in these two regions, its association with tubular structures, likely endocytic structures and the protein is involved in endocytic membrane-trafficking together show that MYO6 is involved in the transport of actin-filament vesicles from the apical region of IHCs to ribbon synapses and/or the retrieval of IHCs synaptic vesicles after exocytosis [21–23]. Moreover, we found that I_Ca_ in Myo6^−/−^ mature mice has a significantly negative V_half_ and a steeper *k*_slope_, showing a hyperpolarizing tendency and stronger voltage-dependence in Ca^2+^ current activation which function synergistically to bring substantially more Ca^2+^ influx the cell under physiological conditions (Figs. 3C, D and 4B). Excessive calcium inflow can lead to an imbalance of Ca^2+^ homeostasis, thus activating a variety of kinases, destroying cytoskeleton and axons, causing energy metabolism and material metabolism disorders, eventually resulting in IHCs impaired, which is directly related to hearing loss [24, 25].

Unlike the MYO6 knock-out mice, the synaptic transmission function of the *Myo6*^*C442Y*^ point mutation developing mice has not significantly discrepancy compared with that of the wild-type. But we found that *Myo6*^*C442Y/C442Y*^ mature mice has remarkably less Ca^2+^ influx the IHCs, down-regulation of ΔC_m_ and lower efficiency of Ca^2+^ triggering synaptic vesicles release for stimulation duration 500 ms. (Fig. 8B–D). Hearing depends on the faithful transmission of auditory signals from IHC to SGN through ribbon synapses [26]. At hair cell afferent synapses, a synaptic ribbon is affiliated with the sites of Ca^2+^ channel clustering and exocytosis. And voltage-gated Ca^2+^ channels are activated according to the graded membrane potential in IHCs and regulate the exocytosis of synaptic vesicles [27]. Changes in the number or characteristics of calcium channels may affect the release of synaptic vesicles of IHCs, which change the synaptic transmission function and eventually lead to hearing loss. Some studies provided clues indicating that vesicle pool replenishment is influenced by Ca^2+^ and depends on a Ca^2+^-sensor protein unique to hair cells called otoferlin [28], which is roughly consistent with our results testifying that *Myo6*^*C442Y*^ point mutation mature mice IHCs have the comparative weak function of exocytosis. MYO6 is directly involved in cargo transport in many cell types [5], and it has been shown to be expressed in the basal pole of IHCs where ribbon synapses are located [10]. It is therefore likely that MYO6 is involved in the maturation, recycling and priming of vesicles. Specifically, Cys^442^ is located at the end of an α-helix that connects the nucleotide-binding structure [29], so that it is not surprising that a mutation at this site change the cargo transport speed of MYO6, ultimately affecting exocytosis at ribbon synapses.

In conclusion, our results suggest that compared with wild-type mice, the ribbon synaptic transmission function of IHCs from different Myosin VI mutant mice is down-regulated, further revealing the physiological and pathological mechanism of Myosin VI in hearing and deafness.

## Materials and methods

### Animals

All experimental procedures described here met the National Institutes of Health guidelines for the Care and Use of Laboratory Animals and were approved by the animal care committee of Fudan University. All mice (both male and female), both developing (age of 8 ~ 10 days) and mature (age of 18 ~ 20 days) were housed on a 12-h light/dark cycle with standard food and water provided ad libitum. We tried our best to minimize the number of animals and their suffering during this study. Sample size and inclusion/exclusion criteria were determined based on previous studies and the results of this study. And no randomization and blinding were used to determine how animals were allocated to experimental groups and processed.

*Myo6*^*C442Y/WT*^, *Myo6*^*C442Y/C442Y*^ and *Myo6*^*WT/WT*^ (control) mice were established by Beijing Biocytogen. Cas9 mRNA and sgRNA were microinjected into fertilized C57BL/6 J oocytes together with a targeting vector containing the C442Y allele to generate F0 founders. Myo6-C442Y mice were crossed with CBA/CaJ mice. Choose progenies without Cdh23ahl, because C57BL/6 J mice have age-related hearing loss due to homozygous Cdh23ahl alleles. Male and female Myo6-C442Y heterozygous mice were crossed to breed WT, heterozygous and homozygous offspring.

*Myo6*^*+/*−^, *Myo6*^−*/*−^ and *Myo6*^*WT/WT*^ (control) mice were prepared using the EGE system developed by Biocytogen based on CRISPR/Cas9. By analyzing the structure of EGE-GJ-033 gene, Exon5 can be knocked out. The sgRNAs were designed in the nonconservative regions of Intron4 and Intron5, respectively, so as to achieve the goal of EGE-GJ-033 gene knockout.

### Electrophysiological recordings

Recordings were achieved at room temperature (20–25 °C). To examine the function of inner hair cells, membrane currents and membrane capacitance (Cm) of IHCs from the apical turn of the sensory epithelium were recorded by conventional whole-cell patch-clamp techniques. Observe the IHCs through a 60×water-immersion objective on the Olympus microscope, and use an EPC10/2 amplifier (HEKA Electronics, Lambrecht Pfalz, Germany) driven by the Patchmaster software (HEKA Electronics) executing patch-clamp recordings. The hair cells were held at −90 mV (millivolt). The liquid junction potential of −10 mV was corrected offline and data were corrected subtracting 10 mV from all potentials.

The apical turns of cochlea were bathed in an oxygenated extracellular solution containing (in mM): 125 NaCl, 10 HEPES, 5.8 KCl, 5.6 D-glucose, 5 CaCl_2_, 0.9 MgCl_2_, 0.7 NaH_2_PO_4_·H_2_O, and 2 Na-pyruvate, pH 7.2 adjusted with NaOH, 300 mOsm/L with NaCl. Patch pipettes coated with dental wax had resistance of 5–6 MΩ full of internal solutions containing (in mM): 135 Cs-methane sulfonate, 10 CsCl, 10 TEA-Cl, 10 HEPES, 3 Mg-ATP, 2 EGTA and 0.5 Na-GTP, pH 7.2 adjusted with NaOH, 290 mOsm/L.

To record Ca^2+^ currents, we applied a slow-rising ramp voltage stimulation from −90 mV to +70 mV to induce whole-cell Ca^2+^ currents, and the peak of this Ca^2+^ currents (I_Ca_) was determined. And recording whole-cell Ca^2+^ currents under different voltages obtained the current-voltage curve (I-V curve) and fitted to the Boltzmann equation:

$${{{\mathrm{I}}}}\left( {{{\mathrm{V}}}} \right) = \left( {{{{\mathrm{V}}}} - {{{\mathrm{V}}}}_{{{{\mathrm{eq}}}}}} \right) \cdot \frac{{{{{\mathrm{G}}}}_{\max }}}{{1 + \exp \left( { - \left( {{{{\mathrm{V}}}} - {{{\mathrm{V}}}}_{1/2}} \right)/k} \right)}}$$

half-activation voltage (V_half_) and the slope factor (*k*) reflecting the steepness of voltage dependence in Ca^2+^ currents activation were obtained from different cells, averaged within the group, and then statistically compared between groups.

In order to quantify the synaptic vesicle release on IHCs, we will apply voltage stimulation to induce the vesicle release, and set a small sine wave voltage stimulation before and after the voltage stimulation. The whole cell membrane capacitance (C_m_) can be measured according to the current response of the cell passive membrane characteristics to sine wave voltage stimulation, and the change of the C_m_ before and after stimulation can measure the exocytosis of synaptic vesicles from IHCs. We fixed the stimulation voltage at −10 mV, and applied 2, 5, 10, 20, 50, 100, 200 and 500 ms (millisecond) to obtain the vesicle release-time data (ΔC_m_-t curve). Then we will use the simple exponential and linear mixed equation [30]:

$${\Delta}{{{\mathrm{C}}}}_{{{\mathrm{m}}}}\left( {{{\mathrm{t}}}} \right) = {{{\mathrm{C}}}}_{{{{\mathrm{m}}}},{{{\mathrm{RRP}}}}} \cdot \left( {1 - \exp \left( {\frac{{{{\mathrm{t}}}}}{{\tau _{{{{\mathrm{RRP}}}}}}}} \right)} \right) + {{{\mathrm{R}}}}_{{{{\mathrm{sustained}}}}} \cdot {{{\mathrm{t}}}}$$

a single exponential function for release of readily releasable pool (RRP) of synaptic vesicles (C_m,RRP_, τ_RRP_) and a linear function for sustained release of synaptic vesicles (R_sustained_). Then the numbers of synaptic vesicles were estimated with the capacitance values, using a conversion factor of 37 aF/vesicle [19].

### Data analysis

Data analysis was performed using Igor 4.0 software (WaveMetrics, Lake Oswego, OR, USA) and GraphPad Prism software 5.0 (GraphPad Software, La Jolla, CA). The data meet the normal distribution. And the variances between the groups being statistically compared are similar. Data are presented as mean ± SEM. Apply one-way and two-way ANOVA with Bonferroni’s post-hoc test (multiple comparisons). *P* value less than 0.05 was deemed significant.

## Data Availability

The datasets used and/or analyzed during the current study are available from the corresponding author on reasonable request.
